# COVID-19 Infection and Circulating ACE2 Levels: Protective Role in Women and Children

**DOI:** 10.3389/fped.2020.00206

**Published:** 2020-04-23

**Authors:** Elena Ciaglia, Carmine Vecchione, Annibale Alessandro Puca

**Affiliations:** ^1^Department of Medicine, Surgery and Dentistry Scuola Medica Salernitana, University of Salerno, Salerno, Italy; ^2^Department of Vascular Physiopathology, IRCCS Neuromed, Pozzilli, Italy; ^3^Cardiovascular Research Unit, IRCCS MultiMedica, Milan, Italy

**Keywords:** infection-immunology, biomarker, screening tools, SARS-CoV, ACE-angiotensin-converting enzyme

It has been reported that angiotensin-converting enzyme 2 (ACE2) is the main host cell receptor of human pathogenic coronaviruses [severe acute respiratory syndrome coronavirus (SARS-CoV), HCoV-NL63, and SARSCoV-2 (COVID-19)], and it plays a crucial role in the entry of virus into the cell to cause the final infection (1). Wrapp and colleagues recently provided the Cryo-EM structure of the virus spike protein, the known ligand for ACE2, and documented a 10 to 20-fold higher affinity of ACE2 for SARS-CoV-2 compared to previous SARS-CoV (2).

ACE2 is mainly expressed by epithelial cells of the lung, intestine, kidney, and blood vessels (3). This may explain the high incidence of pneumonia and bronchitis in those with a severe COVID-19 infection. A recent study showed that ACE2 is also highly expressed on the mucosa of the oral cavity, granting the virus easy access to a new susceptible host (4).

While it has been consolidated that human pathogenic coronaviruses use this same keyhole to enter cells (4, 5), new incoming evidence might suggest that we look at ACE2 as an *ally* in the global fight against COVID-19 fighting rather than an obvious molecular target.

Seen its role as *in vivo* SARS receptor, ACE2 expression was shown to correlate with susceptibility to SARS-CoV spike protein (SARS-S)-driven entry (6, 7), and pathologic alterations in lungs were significantly reduced in ACE2 mutant mice. As a consequence, the systemic treatment with recombinant ACE2 was able to reduce lung injury (8).

On the other hand, ACE2 receptor abundance goes down in the elderly in all these tissues, but, counterintuitively, this might place them at a greater risk of severe illness. So, what of the role of ACE2 in new COVID-19 infection?

The explanation behind this apparent paradox might lie in the post-translational events regulating protein levels and their balance between the membrane-bound and soluble forms. Indeed, ACE2 can undergo an ADAM17 (*a disintegrin and metalloproteinase 17*)-mediated “shedding” from endothelial cells, resulting in the release of the ectodomain with a catalytic and bioactive power into the circulation (9).

Accordingly, in 2014, study scientists found that the circulating ACE2 enzyme offers protection against influenza A (H7N9) virus-induced acute lung injury (10). Some patients with better outcomes have exhibited higher levels of the protein in their sera; meanwhile, turning off the gene for ACE2 led to severe lung damage in mice infected with H5N1, while treating mice with human ACE2 dampened lung injury (10). Furthermore, a single dose of recombinant human ACE2 (GSK2586881; 0.2 mg·kg−1 or 0.4 mg·kg−1 i.v., NCT01884051) has been shown to demonstrate haemodynamic benefits in pulmonary arterial hypertension both in a preclinical and clinical setting (11).

Some previous studies suggested that genetic variants in the ACE2 gene might have a potential to affect ACE2 level in the human body. In the Leeds Family Study, ACE, ACE2, and neutral endopeptidase (NEP) activities were measured in plasma from 534 subjects, and it was indicated that up to 67% of the phenotypic variation in circulating ACE2 could be accounted for by genetic factors (12). Among different polymorphisms, it has been speculated that ACE2 rs2106809 might exhibit primary effects on the ACE2 levels. The circulating ACE2 levels tend to be greater in CC or CT genotype compared with that in the TT genotype. One possible mechanism can be mediated by microRNA, which could modulate endothelial function via translational repression and/or posttranscriptional degradation.

Furthermore, several significant differences in the frequency of distribution of ACE2 variants among different racial and ethnic lines have been described. A recent single-cell RNA-sequencing (RNA-seq) analysis indicated that Asian males may have a higher expression of tissue ACE2 (13). In another case control study conducted in the north eastern Chinese Han population, the serum ACE2 activity negatively correlated with body mass index (BMI), pulse pressure, and estrogen levels in female EH (essential hypertension) patients (14). These observations point both to a cardiovascular protective effect of circulating levels of ACE2 and simultaneously prove that estrogens participate in the upregulation of ACE2 expression and activity levels (15). This might explain the relative protection of female vs. male in COVID-19 infection. Taken together, this evidence seem to indicate that the putative sex predisposition to COVID-19, with men being more susceptible, might be reflective of a peculiar ACE plasma profile.

A putative trend toward this kind of association was also seen in children. Children generally have higher levels of ACE2 than adults (16). For example, ACE levels in children (6 months to 17 years of age) are 13–100 U/l compared with 9–67 U/l in adults when using an FAPGG-based enzymatic activity assay. Of note is the fact that children with confirmed COVID-19 have generally presented with mild symptoms. Cases of coronavirus disease 2019 (COVID-19) among children in China have been less severe than those in adults, according to a new study. In a study of 1,099 patients in China, just 0.9 percent of the confirmed cases were under the age of nine, while only 1.2 percent were between 10 and 19 years old (17). A similar phenomenon in a mouse study in North Carolina was registered by Baric et al.—although SARS-CoV can replicate fairly well, younger animals are really resistant to infection in terms of the disease. When older animals were tested, the severity of SARS illnesses rose (18).

In our opinion, the explanation for the correlation between age and COVID-19 disease severity might be related not only to the immune decline of an aged immune system (termed *immunesenescence*) but also to a peculiar ACE plasma profile that may characterize children from birth. Indeed in mid to late pregnancy in women, an increase in urine and plasma levels of ACE2 were found as well as an increase in local placental/uterine production and activity of ACE2, suggesting a systemic hemodynamic role in the enhancement of placental–fetal blood flow and rapid fetal growth (19).

ACE can pass through the placenta, enabling the mother to transfer to baby her immunity and other kinds of protective soluble factors.

Epidemiological characteristics and transmission patterns of pediatric patients with COVID-19 in China revealed that, contrary to adults, there was no significant gender difference in young patients (20); this is probably due to the influence of the degree of sexual maturation in children and adolescents. Indeed, not only estradiol, via the ER, is a known modulator of the ACE/ACE2 and AT1/AT2 receptor, but ACE is also connected to male reproduction. Catalytic activity of testis ACE contains only the carboxy-terminal domain of ACE, which has exhibited unknown effects on a substrate other than angiotensin I (21).

The reason why the disease is less robust in extremely young animals or humans than in older ones may therefore lie not only in some “cross-immunity” offered by previous infection to “common cold” viruses experienced by children, nor does it lie exclusively in a powerful immune system that, as a result, is not affected by the senescence process; it is probably also affected by an unique ACE2 plasma profile that need to be dissected. By a buffering effect, and much like neutralizing antibodies, soluble ACE2 may help children and asymptomatic people to better counteract virus spreading to a cell target. On one hand, this could help them to contain infection. On the other hand, this could also let these carriers be an important reservoir of circulating virus, and so this deserve much of our attention in the near future.

Answering questions about coronavirus in children and in people who develop less severe symptoms could reverberate well-beyond this escaper population. It could shed light on the reasons why some patients are most at risk and why others could better counteract the spreading of the virus. Furthermore, studying the physiology of those who are less affected could be of help in the development of treatment and a vaccine.

In the last years, the ACE2 activity level has been a potential biomarker for the variations of blood pressure, providing useful information for the prediction and prevention of cardiac dysfunction. Now, circulating level of ACE2 may have prognostic effect in monitoring COVID-infection, and the genetic analysis of ACE2 polymorphisms might be a key element of individualized care for its prevention, diagnosis, and treatment. In this context, an ELISA-based accurate quantification of human soluble ACE2, not only in serum and EDTA plasma but also in more accessible human body fluids (e.g., *saliva, urine, tears, and milk*), should be proposed as a first rapid test screening. To be noted, a standardized protocol for sampling, transport, and storage before its dosage, must be rigorously followed to ensure the accuracy and reliability of inter- and intra-individual quantitation during the course of pathology. Furthermore, correct tests should be carried out in simultaneously in aged-matched healthy volunteers for comparisons. If the current hypothesis is correct, ACE2 determination, by both ELISA and more sensitive HPLC-MS methods, may represent a less extensive and time-consuming means to monitor COVID-19 infection both at pre-clinical and clinical levels.

With the rapid progress that has been made with diagnostic reagents (e.g., nucleic acid and IgM or IgG detection or both), drug repurposing (e.g., remdesivir and chloroquine), immunotherapeutic approaches (e.g., Tocilizumab), and vaccine production as a consequence of the outbreak of novel COVID-19, we thought that it is timely to shed light on the putative link between circulating ACE2 and disease severity. Indeed, as discussed, it may represent a rapidly emerging field of study for therapeutic intervention in the context of COVID-19 infection.

Concerning this, as Penniger JM and colleagues declared in the last days, the availability of recombinant ACE2 (rhACE2; APN01, GSK2586881), its safety profile, and the anti-inflammatory effects (mainly linked to its ability to reduce IL-6 plasma levels) will be the impetus to rapidly launch a pilot trial of rhACE2 as a hopeful treatment option for patients with severe COVID-19 (clinical trials.gov#NCT04287686).

## Author Contributions

All authors listed have made a substantial, direct and intellectual contribution to the work, and approved it for publication.

## Conflict of Interest

The authors declare that the research was conducted in the absence of any commercial or financial relationships that could be construed as a potential conflict of interest.
